# Why We Need Patients and Community at the Center of AI Health Communication Research

**DOI:** 10.2196/97577

**Published:** 2026-05-07

**Authors:** Julie Ayre, Lexuan Shao, Adam G Dunn

**Affiliations:** 1 Sydney Health Literacy Lab, Sydney School of Public Health, Faculty of Medicine and Health, The University of Sydney, Edward Ford Building (A27), Sydney, New South Wales, 2006, Australia; 2 Sydney School of Public Health, Faculty of Medicine and Health, The University of Sydney, Sydney, New South Wales, Australia

**Keywords:** artificial intelligence, health communication, health literacy, patient education, discharge communication

## Abstract

Holderried and colleagues tested whether artificial intelligence (AI)–generated, patient-centered information can help people understand what they need to do after being discharged from a hospital. Participants demonstrated stronger comprehension when they viewed the simplified, patient-centered information rather than a standard letter. This work adds to the available early-phase evidence of AI supporting hospital discharge communication. To meaningfully progress this area of research, we now need to carefully consider how to enhance the design and evaluation of patient-facing AI health communication tools. In this commentary, we argue that equity and consumer engagement remain underrepresented in studies on patient-facing AI health communication tools and describe possible approaches to address this issue.

## Introduction

Artificial intelligence (AI) has great potential to support effective health communication in complex environments, such as hospital discharge [1]. The challenge is clear: hospitals are often unfamiliar places, and patients try to simultaneously manage stress, anxiety, and fatigue while learning about their medical situation and what they need to do next. Miscommunication at discharge can have serious consequences, including readmission to the hospital or even death, and these risks are greater for health priority groups, such as people with low health literacy [2]. Meta-analyses suggest that discharge communication often fails to meet patient needs, with only 47% of patients in emergency departments correctly recalling verbal advice about discharge and only 58% correctly recalling written advice [3]. If AI can meaningfully improve discharge communication, this scalable technology stands to benefit millions of people and the health systems that serve them.

Holderried et al [1] used a novel experimental design to test whether AI-generated, patient-centered information can help people understand what they need to do after leaving a hospital. Participants showed stronger comprehension when they viewed simplified, patient-centered information rather than a standard letter. The study contributes to a growing body of evidence suggesting that AI could meaningfully contribute to more effective communication during this important transition of care [4,5], and it provides a useful example of what a patient-centered evaluation might look like in practice. In this commentary, we discuss the importance of this recently published study and discuss how a stronger focus on equity and consumer engagement can help shift future research further along the pathway toward real-world implementation.

## Start With Evidence-Based Health Literacy Practices

Health literacy is at the heart of equitable communication practices. However, to date, research on AI for patient communication has focused on evaluating accuracy and safety, with limited examples of health literacy evaluation. In the Holderried et al [1] study, patient-centered discharge letters generated by AI scored more favorably on readability than the original discharge letters [6]. Readability is a widely used outcome for estimating the complexity of health information [7]. Despite the improvement in AI-generated letters, readability was still at a level that most people find difficult to understand. This finding is consistent with a recent review of 47 studies showing that although AI tools can significantly improve the readability of health information, prompts to date often fail to produce outputs that meet established readability targets [7]. This may reflect limitations of the AI models themselves and the need for more effective prompts.

Most plain language prompts for AI-generated health advice primarily rely on readability scores to benchmark performance [7]. This approach fails to recognize that readability is a narrow construct that should be supported by other complementary plain language outcomes [8]. Holderried et al [1] recognized this limitation and therefore also assessed the use of medical jargon and acronyms. Researchers could also consider other relevant linguistic features, such as passive voice, vocabulary size, lexical density, and textual cohesion [9]. Beyond automated measures, validated tools, such as the Patient Education Materials Assessment Tool, support a more holistic evaluation, including whether health advice is easy to act on [10]. Discharge letters may also need strategies that are more specific to their content. For example, the Universal Medication Schedule is an established format for medicine instructions that can improve medication adherence, particularly for older patients and people with lower health literacy [11].

## Diverse Consumer Engagement Is Essential

Research stands to benefit from authentic partnerships with diverse patients, carers, and communities and from methodological approaches that embed these voices throughout the research process. For example, consumer engagement can help a research team understand what matters most to patients at discharge, how these needs might vary, and the implications for the design of AI-generated discharge letters and their subsequent evaluation.

In the Holderried et al [1] study, patient actors were trained to be “standardized patients.” Although this is encouraging, given that so few studies of this nature include any consumer or patient perspective [7], there is a clear need to more meaningfully involve consumers with relevant lived experience throughout the research process. This could include early discussions about preferred formats, such as interactive chat, video, or voice AI, and important discussions about when AI advice is not appropriate. Discussions about grounding AI chatbot responses in external knowledge sources (eg, via retrieval-augmented generation) are highly relevant, as these approaches afford greater interactivity and tailoring for patients but also limit the opportunity for human oversight, which poses a potential risk to safety. Lastly, as evidence of an AI tool’s benefits accumulates, studies must seek to evaluate the tool with real patients and in more realistic contexts.

To avoid exacerbating health inequities, researchers should also partner with consumers and stakeholders from priority groups who stand to benefit the most from AI discharge communication tools. These groups may include, for example, patients from culturally and linguistically diverse communities, First Nations communities, people living with severe mental illness or chronic health conditions, older adults, and people with intellectual disabilities [2].

## We Need Frameworks to Guide Decisions About Balancing Risk and Opportunity

As Holderried et al [1] discuss, their AI-generated discharge summaries were not perfect, and five of the learning objectives from the original summaries could not be evaluated because they had been omitted in the patient-centered forms. Although research continues to evaluate the safety and accuracy of clinical AI tools, we also need frameworks to guide decisions about which tools to implement and how to feasibly and ethically do so, including appropriate evaluation methods. These decisions should involve diverse consumers and representative bodies to ensure that judgments about acceptable risk and appropriate use reflect patient needs and priorities. Discussions can be further supported by data reporting on the fairness and equity of these AI tools.

## Conclusion

Holderried et al [1] provide new evidence showing the feasibility of an AI tool for improving discharge communication. This is one of few published examples where evaluation methods incorporate multiple health literacy assessments and explore learning outcomes. Now is the time to design studies that make full use of evidence-based health literacy practices and meaningfully involve diverse consumers throughout the research process. This is critical for ensuring that research meets patient needs and meaningfully improves the health care experience.

## Abbreviations

AI: artificial intelligence

## References

[1] Holderried F, Sonanini A, Stegemann-Philipps C (2026). Impact of GPT-4-generated discharge letters on patients’ medical comprehension: prospective crossover study. J Med Internet Res.

[2] Berkman ND, Sheridan SL, Donahue KE, Halpern DJ, Crotty K (2011). Low health literacy and health outcomes: an updated systematic review. Ann Intern Med.

[3] Hoek AE, Anker SCP, van Beeck EF, Burdorf A, Rood PPM, Haagsma JA (2020). Patient discharge instructions in the emergency department and their effects on comprehension and recall of discharge instructions: a systematic review and meta-analysis. Ann Emerg Med.

[4] Kumar A, Wang H, Muir KW, Mishra V, Engelhard M (2025). A cross-sectional study of GPT-4–based plain language translation of clinical notes to improve patient comprehension of disease course and management. NEJM AI.

[5] Stanceski K, Zhong S, Zhang X (2024). The quality and safety of using generative AI to produce patient-centred discharge instructions. NPJ Digit Med.

[6] Eisinger F, Holderried F, Mahling M (2025). What’s going on with me and how can I better manage my health? The potential of GPT-4 to transform discharge letters into patient-centered letters to enhance patient safety: prospective, exploratory study. J Med Internet Res.

[7] Ugas M, Huynh J, Lenarcik-Packham A (2026). The utility of artificial intelligence in plain language writing: a scoping review. Patient Educ Couns.

[8] Mac O, Muscat DM, Ayre J, McCaffery K (2025). Fundamentally flawed or functional and feasible? The use of readability metrics in healthcare. Patient Educ Couns.

[9] Ayre J, Bonner C, Muscat DM (2023). Multiple automated health literacy assessments of written health information: development of the SHeLL (Sydney Health Literacy Lab) Health Literacy Editor v1. JMIR Form Res.

[10] Shoemaker SJ, Wolf MS, Brach C (2014). Development of the Patient Education Materials Assessment Tool (PEMAT): a new measure of understandability and actionability for print and audiovisual patient information. Patient Educ Couns.

[11] Davis TC, Federman AD, Bass PF (2009). Improving patient understanding of prescription drug label instructions. J Gen Intern Med.

